# Letter to the Editor on “Urinary heavy metal burden and overactive bladder risk: a cross-sectional study based on NHANES 2005–2018”

**DOI:** 10.1097/JS9.0000000000004774

**Published:** 2026-01-13

**Authors:** Yanqin Song

**Affiliations:** Reproductive Medicine Center, Hainan Women and Children’s Medical Center, Haikou, China

*Dear Editor,*

Wang *et al* analyze seven NHANES cycles (2005–2018; *n* = 9086) to evaluate whether urinary heavy metals – considered individually and as mixtures – are associated with overactive bladder (OAB)[1]. This question is clinically relevant because OAB affects a substantial proportion of adults and imposes a marked quality-of-life and health-system burden[2].

A key strength is the authors’ “triangulation” across complementary methods. After creatinine correction and ln-transformation of urinary metals, they combine multivariable logistic regression with non-linear modeling and two mixture approaches [weighted quantile sum (WQS) and quantile g-computation]^[1,3]^. The main signal is consistent: cadmium (Cd) emerges as the dominant positive driver of OAB risk across single-metal models and mixture analyses, with additional contributions from lead (Pb) and uranium (Ur)[1]. This aligns with the direction of recent NHANES-based evidence linking urinary Cd to OAB risk, supporting external coherence of the Cd finding[4].

The “negative-direction” mixture findings are more nuanced and deserve careful framing. When the mixture effect is constrained to be protective, barium (Ba) carries the largest negative weight and higher WQS quartiles associate with lower OAB odds[1]. Given that WQS is direction-constrained by design, such bidirectionality (Cd-driven positive mixture vs Ba-driven negative mixture) is best interpreted as hypothesis-generating rather than as evidence that Ba is inherently protective^[1,3]^. The reported non-linearities (e.g., U-shaped patterns for Ba and thallium) further reinforce that simple “more is better/ worse” messaging is inappropriate[1].

Several limitations temper causal interpretation. The cross-sectional design cannot establish temporality, and reverse causation is plausible: LUTS/OAB-related changes in hydration, comorbidity, or renal handling may influence urinary metal concentrations. Single spot-urine measures may misclassify longer-term exposure despite creatinine adjustment[1]. In addition, OAB is questionnaire-defined rather than clinically adjudicated, and residual confounding from diet, occupation, smoking intensity, and co-exposures may persist. Finally, while guidelines emphasize structured diagnostic assessment for OAB, translating environmental association signals into clinical pathways will require prospective confirmation and better exposure-source resolution[5].

Overall, Wang *et al* provide a valuable population-level signal that urinary heavy metal mixtures – particularly Cd – are associated with OAB risk[1]. Future longitudinal studies with repeated exposure measures and validated clinical phenotyping are needed to test causality and to identify actionable prevention targets. This commentary was prepared in line with the TITAN 2025 guideline to promote transparency and accountability in reporting[6].

## Data Availability

No data was used in this Letter to the Editor.
